# Analysis on quality of life, anxiety, and depression of diquat self-poisoning after returning to the community: a follow-up study

**DOI:** 10.3389/fpubh.2026.1940440

**Published:** 2026-09-09

**Authors:** Zejin Ou, Jifeng Dai, Caiying Zhong, Shi Chen, Ling Zhou, Yufan Deng, Meiyi He, Yuquan Chen, Yuxia Zhang, Zhi Wang

**Affiliations:** 1 Key Laboratory of Occupational Environment and Health, Guangzhou Twelfth People's Hospital, Guangzhou, China; 2 Institute of Occupational and Environmental Health, Guangzhou Medical University, Guangzhou, China; 3 School of Public Health, North Sichuan Medical College, Nanchong, China; 4 School of Public Health, Southern Medical University, Guangzhou, China; 5 College of Traditional Chinese Medicine, Guangdong Pharmaceutical University, Guangzhou, China; 6 Department of Occupational Diseases and Poisoning, Guangzhou Twelfth People's Hospital, Guangzhou, China

**Keywords:** anxiety, depression, diquat, follow-up, quality of life, self-poisoning

## Abstract

**Objective:**

Pesticide self-poisoning is a major global public health issue, yet the post-discharge mental health of survivors remains poorly characterized. To investigate the physical and mental health among survivors of diquat self-poisoning after returning to the community, which informs the effective prevention and intervention measures of repeated suicide.

**Methods:**

A follow-up study of 25 patients with acute diquat self-poisoning was enrolled from Guangzhou twelfth people’s hospital during 2017 to 2023. The health-related quality of life (HRQoL) was evaluated using the MOS 36-item short-form health survey (SF-36), and anxiety and depression were, respectively, assessed using Generalized Anxiety Disorder-7 (GAD-7) and ‌Patient Health Questionnaire-9 (PHQ-9) scales.

**Results:**

Twenty-five (25) diquat self-poisoning patients were enrolled in a follow-up study, with a median follow-up of 1.58 person-years. Most participants had low scores in mental health of the HRQoL survey, with a median score was 108.15. In SF-36 domains, only Role Physical (RP) scored significantly lower in the high-dose group (*p* = 0.04). Persistent neurological issues (fatigue, forgetfulness, headache) were the primary physical complaints. HRQoL total scores were significantly shaped by sleep duration, history of major trauma, left-behind child status, and psychological symptoms (*p* < 0.05). Anxiety and depression symptoms had high prevalent rates of 56 and 68%, respectively. Stratified analysis by toxic dose [low-dose (≤ 50 mL, n = 12) vs. high-dose (> 50 mL, n = 13)] revealed no statistically significant differences in anxiety or depression rates.

**Conclusion:**

Diquat self-poisoning survivors may experience persistent physical and psychological health difficulties after returning to the community, warranting further attention. The findings highlighted that prevention of repeated self-harm is still necessary among at-risk populations after returning to the community, particularly adolescents and young adults.

## Highlights

1. This is the first follow-up investigation to systematically assess long-term psychosocial outcomes among diquat self-poisoning survivors upon community reentry, addressing a critical evidence gap.
2. The findings highlights that the low mental score requires effective prevention measures against repeated self-harm in at-risk subjects, particularly adolescents and young adults.
3. The single-central, cross-sectional design with a modest sample size limits statistical power for detecting subtle associations and precludes the establishment of causal trajectories or the tracking of dynamic symptom evolution.

## Introduction

Intentional suicide has been a severe global public health challenge in recent years. More than 700,000 deaths were caused by intentional suicide each year, ranking as the fourth leading cause of death among people aged 15 to 29 years, and an estimated 77% of these deaths occurred in low-and middle-income countries (1). The 12-month prevalence of suicide attempts in young people reached up to 4.2% in some low-resource settings (2). An estimated 280 million people lived with depression and 301 million with anxiety disorders (3), which were among the main risk factors for suicidal behavior (4). However, mental health infrastructure remained profoundly under-resourced in many underdeveloped regions (5).

Pesticide self-poisoning accounted for a substantial proportion of the global suicide toll. Between 2006 and 2015, an estimated 168,000 deaths annually were attributable to intentional pesticide ingestion, representing roughly 19.7% of all suicides worldwide (6). Among adolescents and young adults, this pattern was especially dangerous because they exhibited disproportionately high rates of self-harm repetition and long-term psychiatric morbidity after a medically serious self-poisoning event (7). A study of acute organophosphorus pesticide poisoning survivors documented a persistently high prevalence of anxiety, depression, and cognitive impairment long after discharge, accompanied by a substantially lower health-related quality of life (8). The psychosocial vulnerability of survivors remains largely unaddressed by health systems (9, 10), and the absence of post-discharge mental health support frequently fuels a vicious cycle of repeated self-harm (11). Additionally, legal and administrative barriers, such as mandatory reporting and medico-legal procedures, may contribute to underreporting of pesticide self-poisoning in some settings and can influence survivors’ willingness to seek follow-up care.

A particularly concerning development was the increasing global use of diquat, a bipyridine herbicide, following the strict restriction of paraquat. Acute diquat self-poisoning was characterized by rapid progression to severe multiple organ dysfunction and toxic encephalopathy, with in-hospital mortality rates ranging from 16.7 to 60% in recent cohorts (12, 13). Although intensified clinical protocols improved acute survival, the long-term psychological and functional outcomes of diquat survivors remained entirely unknown. In other forms of pesticide self-poisoning, prospective data showed that survivors endured persistent depressive and anxiety disorders, cognitive complaints, and markedly impaired health-related quality of life years after the initial episode (14). Furthermore, follow-up studies confirmed that individuals presenting with self-poisoning were at sustained elevated risk of fatal repetition (15). However, empirical evidence characterizing the post-discharge health status of diquat self-poisoning patients was lacking, which was necessary to inform the development of tailored aftercare strategies.

Therefore, a follow-up study was designed to investigate the health status of patients following intentional diquat self-poisoning after community reintegration. Standardized psychometric instruments were used to comprehensively evaluate their long-term physical and mental health, providing robust evidence to guide community-based psychological interventions and health management.

## Materials and methods

### Study design and subjects

**Figure 1:**
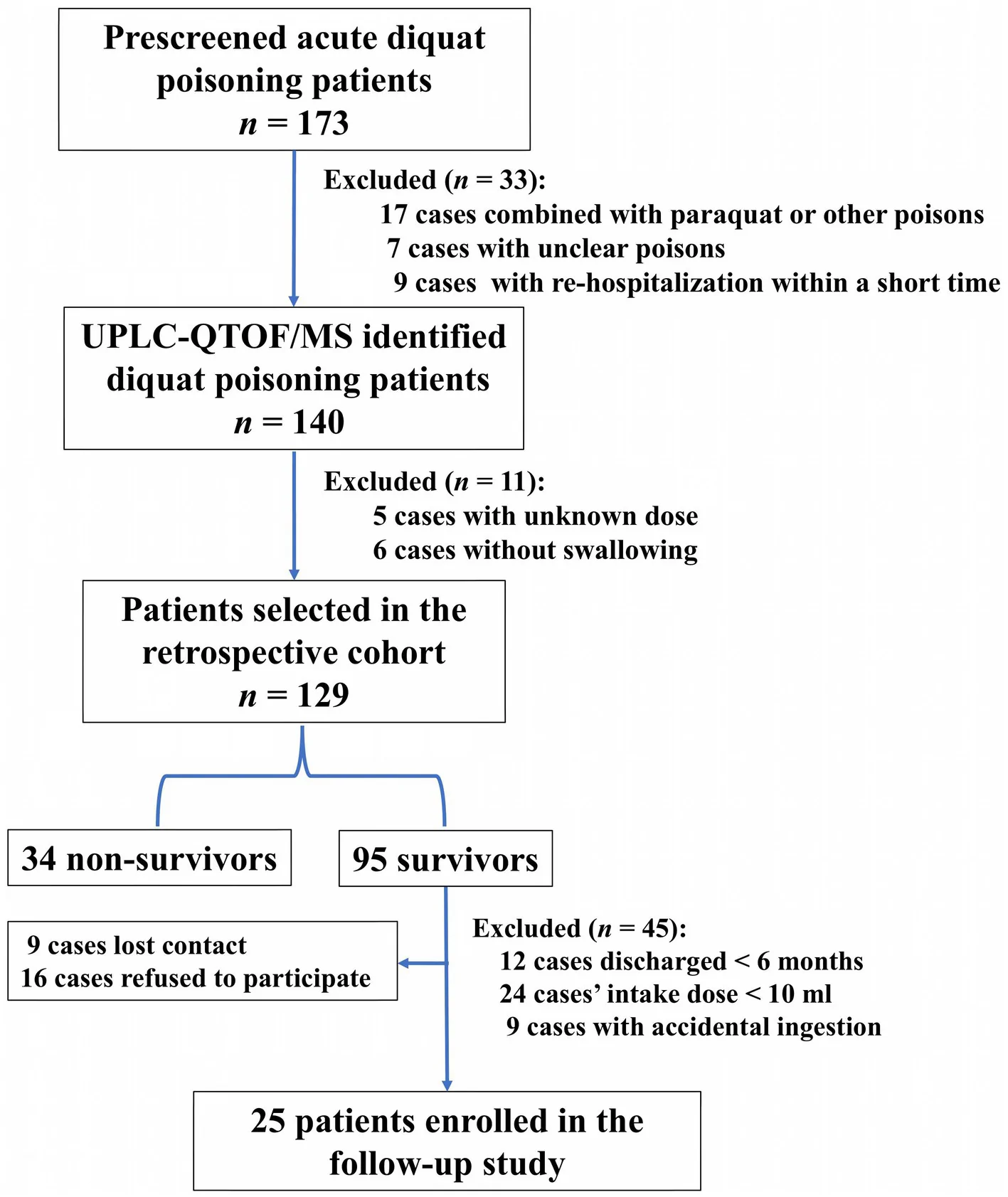
Flow diagram of participant enrollment among acute diquat poisoningsurvivors. Of 129 patients with confirmed acute diquat poisoning, 34 died during hospitalization. After excluding 70 of the 95 survivors, 25 eligible participants completed the follow-upassessment.

This study employed a cross-sectional follow-up design during May 2017 and May 2023. There was a total of 129 patients with confirmed acute diquat poisoning were treated at Guangzhou Twelfth People’s Hospital. The toxins were identified using ultra-high performance liquid chromatography quadrupole-time of flight mass spectrometry (UPLC-QTOF/MS) in blood or urine. Of these, 34 patients died during hospitalization, and 95 patients survived following the treatment. Among those surviving patients, 25 patients met the following inclusion criteria and completed the follow-up assessment: (1) confirmed diagnosis of acute diquat poisoning and successfully discharged; (2) Intentional self-poisoning; (3) estimated ingestion dose ≥ 10 mL; (4) survival post-discharge for more than 6 months; and (5) provision of written informed consent. On the other hand, 70 patients were excluded from the analysis: 12 were discharged from hospital < 6 months, 24 had an intake dose < 10 mL, 9 were accidental ingestion. Additionally, 25 patients declined participation or were lost to follow-up. The participant enrollment process is summarized in Figure 1.

### Assessment tools and data collection

Structured face-to-face or telephone interviews were systematically executed by uniformly trained clinical investigators using a standardized questionnaire.

#### Demographic and clinical profiles

Data encompassing age, gender, suicide motivation, estimated ingestion volume, and persistent clinical symptoms during the follow-up period were thoroughly recorded. An RCS model (16) was utilized to identify inflection points in the risk of severe clinical outcomes, establishing a threshold of 50 mL for stratification. Accordingly, patients were categorized into low-dose (≤ 50 mL) and high-dose (> 50 mL) groups.

#### Health-related quality of life (HRQoL)

HRQoL was evaluated using the MOS 36-item short-form health survey (SF-36), which captures 8 sub-domains compiled into the Physical Component Summary (PCS) and Mental Component Summary (MCS). Higher aggregate scores reflect superior health status (17).

To facilitate clinical interpretation, composite scores were further categorized into three ordinal levels based on the degree of deviation from Chinese population norms (18): “good” (≥117, approximating the normative range), “fair” (81–116, representing mild-to-moderate impairment), and “poor” (≤80, corresponding to severe impairment > 2 standard deviations below the normative mean).

#### Psychological status

General anxiety symptoms were evaluated via the 7-item Generalized Anxiety Disorder scale (GAD-7), with a total score ≥ 5 defined as positive for anxiety (19). Depressive symptoms were screened using the 9-item Patient Health Questionnaire (PHQ-9), with a total score ≥ 10 established as the threshold for clinical depression (20).

### Statistical analysis

Data processing was executed via Excel 2021, and statistical analyses were performed using Python 3.13.2. Continuous variables were presented as median and interquartile range [M (IQR)] and compared using the Mann–Whitney *U* test, as the data were not normally distributed. Categorical data were expressed as frequencies and percentages [*n* (%)] and compared using Fisher’s exact test, which is appropriate for small sample sizes and low expected cell counts. Effect sizes (Cliff’s delta for continuous comparisons and odds ratios with 95% confidence intervals for categorical comparisons) were calculated to quantify the magnitude of differences beyond statistical significance. All statistical verifications were two-sided, with statistical significance defined as *p* < 0.05.

### Patient and public involvement

Patients and the public were not formally involved in the design, conduct, reporting, or dissemination plans of this research. This was primarily due to the acute, life-threatening nature of diquat self-poisoning and the profound post-discharge psychosocial vulnerability of survivors, which rendered the prospective establishment of a patient advisory group unfeasible. However, the selection of patient-reported outcome measures was guided by the commonly voiced concerns (such as fatigue, cognitive complaints, and sleep disturbances) documented in previous poisoning survivor interviews, ensuring the assessment captured domains of relevance to this population. The burden of the questionnaire was consciously limited to 30 min to respect the psychological capacity of participants. Upon completion of the study, all participants will receive a plain-language summary of the aggregated findings, and further efforts will be made to engage community-based mental health organizations and survivor support networks to co-develop future patient-centered interventions aimed at breaking the “treatment-discharge-relapse” cycle.

## Results

### Baseline characteristics of the follow-up cohort

**Figure 2:**
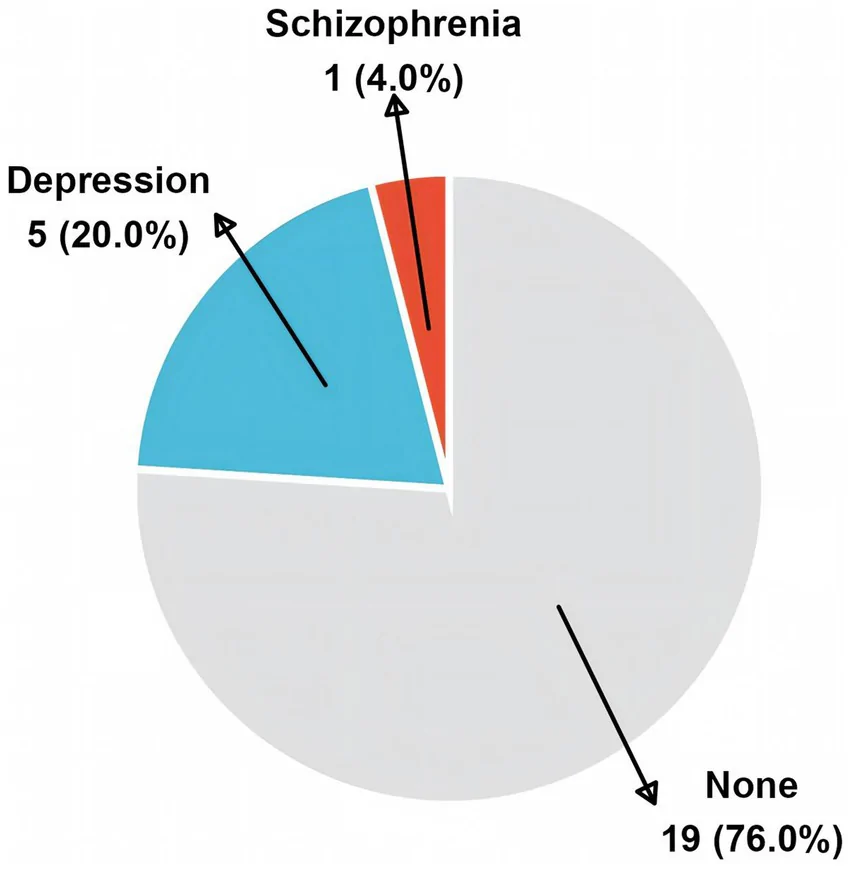
Distribution of previous psychiatric history among acute diquat poisoning survivors. The majority of patients (76%, *n* = 19) reported no prior psychiatric history, while 20% (*n* = 5) had a documented history of depression and 4% (*n* = 1) had a prior diagnosis of schizophrenia.

The comprehensive demographic and baseline clinical characteristics of the cohort were summarized in Table 1. Among the 25 enrolled survivors, the age ranged from 13 to 55 years, with adolescents and young adults under 18 years old constituting 32% (*n* = 8) of the population. Females comprised 60% (*n* = 15) of the cohort. Regarding education, patients with a junior high school education or below accounted for the largest proportion (48%, *n* = 12). Interpersonal and family conflicts were identified as the primary trigger for self-poisoning, accounting for 40% (*n* = 10) of the cases. Additionally, 40% (*n* = 10) of the patients possessed a basic pre-existing understanding of pesticide hazards. Regarding early social determinants, 20% (*n* = 5) of the participants possessed a childhood background of lacking parental companionship (left-behind children). In terms of baseline psychiatric comorbidities, 24% (*n* = 6) of the survivors had an established psychiatric history prior to the incident (20% with depression and 4% with schizophrenia), while the remaining 76% (*n* = 19) reported no previous psychiatric diagnoses (Figure 2).

**Table 1:** Baseline characteristics of survivors of acute diquat poisoning.

Variables		*n* (%)	*p*
Gender	Male	10 (40)	0.11
	Female	15 (60)	
Age at onset	<18	8 (32)	0.84
	≥18	17 (68)	
Education level	Junior high school and below	12 (48)	0.39
	Senior high or vocational school	7 (28)	
	College and above	6(24)	
Marital status	Unmarried	19(76)	0.56
	Married or divorced	6(24)	
Previous occupation	Student	10(40)	
	Manual worker	10(40)	0.25
	Other	5(20)	
Current residence	Rural area or town	6(24)	0.88
	County or small-to-medium city	12(48)	
	Provincial capital	7(28)	
Smoking status	Non-smoker	15(60)	0.64
	Smoker	10(40)	
Alcohol consumption	Non-drinker	16(64)	0.42
	Drinker	6(24)	
	Former drinker	3(12)	
Dietary flavor preference	High-salt diet	7(28)	0.34
	High-fat/high-sugar diet	6(24)	
	Light diet	12(48)	
Dietary pattern	Balanced diet	15(60)	0.39
	Meat-based diet	7(28)	
	Plant-based diet	3(12)	
Physical exercise	Never	12(48)	0.72
	Occasionally	7(28)	
	Frequently	6 (24)	
Average sleep duration	≥8 h	9 (36)	0.00*
	<8 h	16 (64)	
Sleep quality	Good	5 (20)	0.57
	Fair	14 (56)	
	Poor	6 (24)	
Monthly household income	≤5,000	6 (24)	0.08
	5,000–8,000	8 (32)	
	>8,000	11 (44)	
Family structure type	Two-parent family	19 (76)	**
	Single-parent family	4 (16)	
	Absent-parent family	1 (4)	
	Blended family	1 (4)	
Cause of the accident	Family conflicts	10 (40)	0.30
	Romantic relationship issues	5 (25)	
	Mental health issues	5 (25)	
	Other	6 (24)	
Knowledge of pesticides	No knowledge	4 (16)	0.90
	Little knowledge	7 (28)	
	Basic knowledge	10 (40)	
	Familiar	4 (16)	
History of major life events	Yes	4 (16)	0.04*
	No	21 (84)	
Left-behind child status	Yes	5 (20)	0.02*
	No	20 (80)	
Ingestion dose	Low dose	12 (48)	0.72
	High dose	13 (52)	
Rehabilitation modalities	No treatment	9 (36)	0.02*
	Pharmacological therapy	10 (40)	
	Physical therapy	6 (24)	
Frequency of rehabilitation	1–3 sessions	12 (48)	0.08
	≥4 sessions	4 (16)	
Duration of rehabilitation	≤3 months	8 (32)	0.06
	>3 months	8 (32)	
Presence of depression	Yes	17 (68)	0.02*
	No	8 (32)	
Presence of anxiety	Yes	14 (56)	0.01*
	No	11(44)	
Quality of life grade	Good	8 (32)	-
	Fair	16 (64)	
	Poor	1 (4)	

Data are presented as *n* (%). *p*-values were derived from univariate analysis examining associations between each variable and HRQoL total scores. *p < 0.05. **Statistical comparison not performed due to zero cell frequency in certain categories.

Based on the estimated ingestion volume upon admission and blood concentration screening, patients were categorized into a high-dose group (>50 mL; 52%, *n* = 13) and a low-dose group (≤50 mL; 48%, *n* = 12). Upon follow-up, psychometric screenings unveiled a high burden of psychological distress: the overall prevalence of anxiety and depressive symptoms reached 56% (*n* = 14) and 68% (*n* = 17), respectively. The overall HRQoL was rated as “good” in 32% (*n* = 8) and “fair” in 64% (*n* = 16) of the cohort. Furthermore, univariate analysis indicated that average sleep duration, exposure to major traumatic events, childhood left-behind experience, the specific content of rehabilitative therapies, and the presence of comorbid anxiety or depressive symptoms were significantly associated with the overall HRQoL scores (*p* < 0.05).

### Distribution of major clinical symptoms

**Figure 3:**
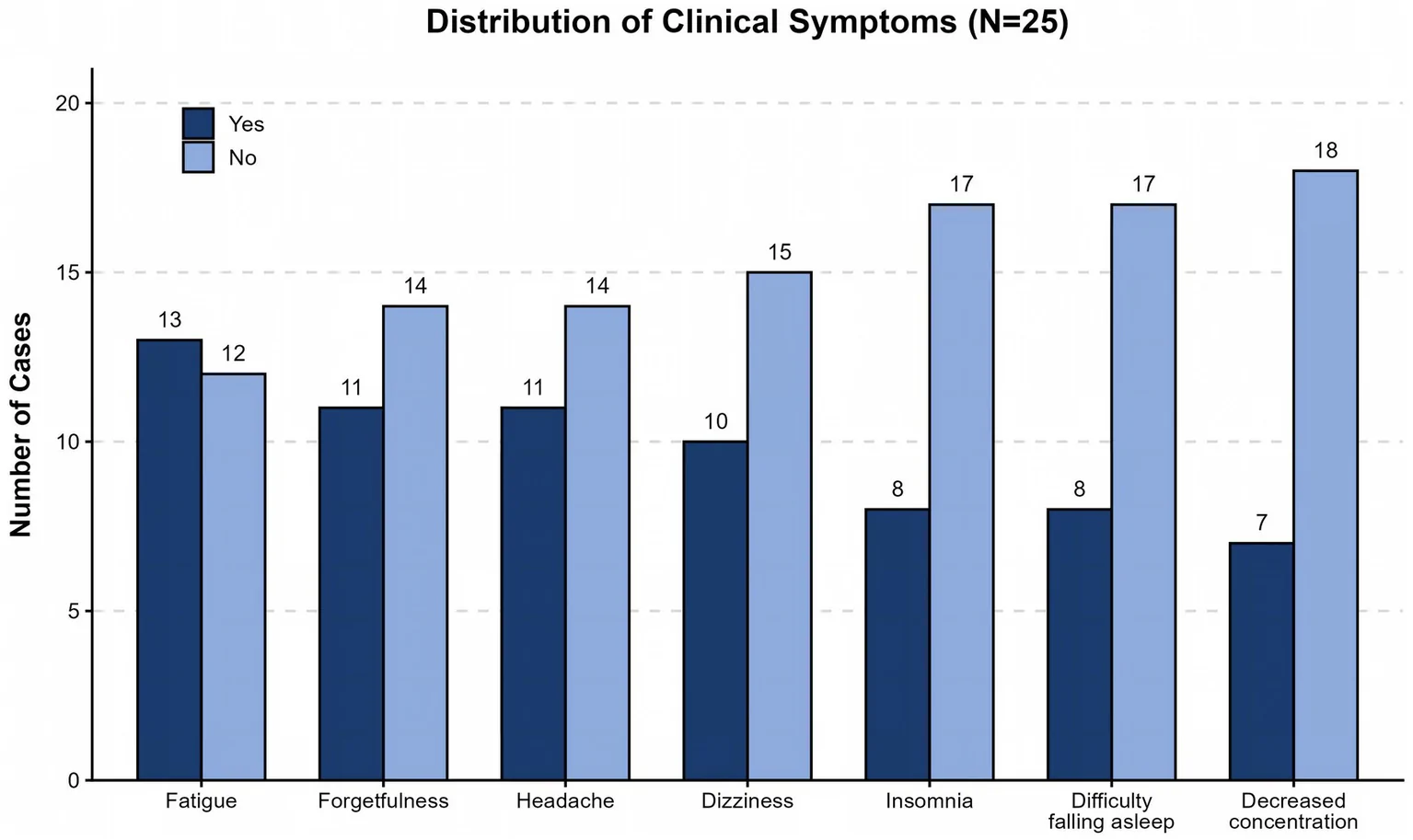
Distribution of major clinical symptoms among survivors of acute diquat poisoning. Symptoms were predominantly neurological in nature, with easy fatigability/weakness (52%), forgetfulness (44%), and headache (44%) being the most prevalentcomplaints.

Upon community reentry, survivors exhibited prominent long-term somatic complaints primarily clustered around central and peripheral neurological impairments. Specifically, easy fatigability and weakness were reported by 52% (*n* = 13) of the patients, forgetfulness by 44% (*n* = 11), headache by 44% (*n* = 11), dizziness by 40% (*n* = 10), initial insomnia/sleep disturbances by 32% (*n* = 8), decreased attention by 28% (*n* = 7), and blurred vision by 12% (*n* = 3). The detailed distribution of these clinical symptoms was illustrated in Figure 3.

### Univariate analysis of HRQoL dimensions by dose stratification

The overall median SF-36 composite score for the 25-patient cohort was 108.15 (interquartile range [IQR]: 97.15–117.40). Based on the norm-referenced categorization, 24 patients (96%) attained either a “good” (*n* = 8, 32%) or “fair” (*n* = 16, 64%) HRQoL rating. The single patient classified as “poor” recorded the lowest composite score in the cohort (71) and concurrently exhibited the lowest Mental Component Summary (MCS) score (19), alongside a Physical Component Summary (PCS) score of 45 (cohort minimum: 44). Notably, the patient with the highest anxiety score (19) also reported depressive symptoms and remained within the “fair” HRQoL range; similarly, the patient with the highest depression score (18) presented with comorbid anxiety and was likewise classified as “fair.”

As shown in Table 2, when comparing HRQoL total scores and individual sub-domains between the low-dose and high-dose groups, the role physical (RP) dimension emerged as the sole metric demonstrating a statistically significant difference (*p* < 0.05). Specifically, the low-dose group maintained a significantly higher median RP score (100; IQR: 68.75–100) compared to the high-dose group (50; IQR: 0–75). The calculated Cliff’s delta (δ) was 0.47, indicating a large effect size and suggesting that ingestion dose may have a substantial impact on role physical functionality. The remaining dimensions, including the total HRQoL score, physical component summary (PCS), mental component summary (MCS), and other individual sub-domains, showed no statistically significant variations between the two dose cohorts (*p* > 0.05).

**Table 2:** Univariate analysis of SF-36 domain scores between the low- and high-dose groups.

Variable	Total (*N* = 25)	Low-dose group (*n* = 12)	High-dose group (*n* = 13)	*p*	Cliff’s Delta (95% CI)
Quality of Life score	108.15 (97.15, 117.40)	111.98 (97.23, 117.55)	103 (97.16, 116)	0.72	0.09 (0.39–0.54)
Physical Component Summary	71.50 (60.50, 87.50)	78.50 (70.13, 89.25)	61 (59.75, 87.50)	0.29	0.26 (0.22–0.69)
Physical Functioning	95 (85, 100)	97.50 (87.50, 100)	90 (85, 100)	0.46	0.17 (0.29–0.61)
Role Physical	75 (25, 100)	100.0 (68.75, 100.0)	50 (0, 75)	0.04*	0.47 (0.08–0.83)
Bodily Pain	84 (74, 100)	79 (62, 100)	84 (74, 100)	0.25	0.27 (0.67–0.87)
General Health	60 (45, 85)	65 (43.75, 81.75)	60 (45, 85)	0.76	0.08 (0.41–0.52)
Mental Component Summary	67.88 (43.88, 77)	70.31 (48.02, 77.13)	55.92 (41.25, 76.75)	0.43	0.19 (0.30–0.62)
Vitality	50 (40, 65)	47.50 (43.75, 67.50)	55 (35, 60)	0.66	0.11 (0.39–0.56)
Social Functioning	75 (62.50, 100)	75 (62.50, 87.50)	87.50 (62.50, 100)	0.69	0.10 (0.57–0.36)
Role Emotional	100 (0, 100)	100 (58.33, 100)	33.33 (0.03, 100)	0.15	0.31 (0.12–0.67)
Mental Health	52 (40, 68)	58 (40, 65)	48 (36, 72)	0.87	0.05 (0.40–0.51)

Data are presented as median (interquartile range [IQR]). *p*-values were derived from Mann–Whitney *U* tests. Effect sizes were quantified using Cliff’s delta (δ) with 95% confidence intervals. *p < 0.05.

### Association of anxiety and depression with ingestion dose

To evaluate potential dose–response trends regarding psychological morbidities, the presence of anxiety and depressive symptoms was compared between the low-dose and high-dose groups. Results indicated no statistically significant differences in the occurrence of anxiety or depression between the two dosage cohorts (*p* > 0.05). Specifically, the odds ratio (OR) for developing anxiety symptoms in relation to a high-dose exposure was 0.43, while the OR for developing depressive symptoms was 2.38 (Table 3). These findings underscored a distinct non-dose-dependent relationship for post-discharge psychological status in this cohort.

**Table 3:** Univariate analysis of anxiety and depression symptoms between the low- and high-dose groups.

Variable	Low-dose group (%)	High-dose group (%)	*p*	OR(95%CI)
Anxiety	66.67	46.15	0.43	0.43(0.08–2.17)
Depression	58.33	53.85	0.41	2.38(0.42–13.39)

Data are presented as percentages. *p*-values were derived from chi-square or Fisher’s exact tests as appropriate. OR, odds ratio; CI, confidence interval.

## Discussion and conclusion

### Principal findings

The present work was the first follow-up study to comprehensively analyze the long-term health outcomes of patients with diquat self-poisoning upon community reentry. Our findings revealed that the post-discharge prevalence of anxiety and depression among these suicide survivors was profoundly elevated compared to both the general public and survivors of non-pesticide-related physical diseases (21). Globally, pesticide self-poisoning accounted for a substantial proportion of all suicides, imposing a profound public health burden (22). Furthermore, the immediate post-discharge period was a highly vulnerable window, as survivors faced a markedly increased risk of repeated self-harm and completed suicide, with meta-analytic evidence demonstrating that prior self-harm history nearly tripled the odds of subsequent suicidal behavior (23). This observed morbidity underscores the limited psychosocial support available to these individuals after acute medical stabilization, frequently stranding them in a vicious “treatment-discharge-relapse” cycle.

### Interpretation and implications

It is essential to acknowledge that the scarcity of long-term psychosocial follow-up studies on diquat self-poisoning survivors-and the modest sample size of the present investigation-stems not merely from the absolute rarity of the condition but from a confluence of methodological and psychosocial barriers that systematically impede participant recruitment. First, the exceptionally high case fatality of diquat self-poisoning fundamentally restricts the pool of eligible survivors. A meta-analysis of Chinese data demonstrated that, while in-hospital mortality stands at approximately 18.6%, cumulative mortality rises to 60% after extended follow-up, indicating that more than half of hospitalized patients ultimately succumb to the toxicity (24). Second, among survivors who re-enter the community, profound stigma associated with suicide attempts forms a formidable barrier to research participation. Qualitative evidence has shown that suicide attempt survivors frequently avoid disclosing their histories or engaging with follow-up studies due to intense fears of negative labeling, social distancing, and moral judgment from family, employers, and the broader community (25). Third, the underreporting of pesticide self-poisoning is well documented in low- and middle-income countries, where legal and administrative barriers-such as mandatory police reporting and medico-legal procedures-further discourage patients and families from seeking hospitalization or disclosing intentional ingestion (26). Each of these factors collectively narrows the pool of survivors available for longitudinal mental health research. Against this backdrop, the successful enrollment of 25 diquat self-poisoning survivors with complete psychosocial assessments constitutes a unique and invaluable dataset, offering the first systematic glimpse into the often-hidden psychiatric burden that afflicts this population upon community reintegration.

Although our data did not reveal a statistically significant dose-dependent relationship for anxiety and depression-likely limited by the small sample size-this phenomenon raised the possibility of a dual etiology driven by distinct biological and psychosocial mechanisms. Biologically, high-dose diquat exposure may exert neurotoxic effects involving oxidative stress and neurotransmitter dysregulation, which could contribute to organic depressive symptoms. Consistent with this possibility, a recent systematic review found that pesticide poisoning nearly tripled the risk of depression compared with non-poisoned individuals (27). This neurobiological pathway was consistent with a growing body of evidence linking acute pesticide poisoning to a substantially elevated risk of depression: a recent systematic review and meta-analysis found that pesticide poisoning nearly tripled the risk of depression compared to non-poisoned individuals, with a pooled odds ratio of 2.94 (95% CI = 1.79–4.83, *p* < 0.001) (21). Beyond emotional regulation, survivors’ core post-discharge complaints-such as fatigue, forgetfulness, and headache-strongly suggested that diquat penetrated the blood–brain barrier to induce widespread dopaminergic neuron damage, triggering oxidative stress and neuroinflammation that manifested directly as chronic cognitive and physical symptoms (28).

Psychosocially, however, low-dose survivors-who maintained full consciousness during the acute phase and escaped crippling organic deficits-faced a severe emotional toll upon community reentry. They were uniquely vulnerable to intense self-perceived stigma, profound shame, and immediate anxieties regarding the sudden economic burdens of their medical care (29). Furthermore, this profound psychological distress aligned with the immense mental burden caused by chronic post-poisoning pain, organ dysfunction, and sudden disruption of social roles (30). In this context, untreated depressive symptoms could also delay physical recovery through complex, detrimental interactions within the “psycho-immune” axis (31). The dual-etiology framework we proposed was further supported by emerging evidence that distinct clinical trajectories existed among pesticide-poisoned patients, wherein biological severity and psychosocial vulnerability interacted to shape heterogeneous long-term outcomes (32). Conversely, the fact that the Role Physical (RP) domain was uniquely dose-dependent indicated that high-dose ingestion left a lasting physical footprint, likely stemming from chronic multi-organ fibrosis or subclinical parenchymal damage (33), which directly restricted patients’ daily activities and impaired their ability to execute occupational and domestic roles due to physical decline (34).

Furthermore, our analysis highlighted that restricted sleep duration synergized with diminished HRQoL (35). Sleep deprivation is known to exacerbate systemic oxidative stress, which creates a destructive feedback loop with diquat-induced free radical pathways, thereby hindering long-term neuro-somatic repair (36). Specifically, sleep disturbances (e.g., difficulty falling asleep) can suppress immune responses, disrupt neuroendocrine homeostasis, and aggravate chronic fatigue and organ damage (37), ultimately driving a devastating “toxicity-sleep disturbance-symptom deterioration” cycle (38). This mechanism was particularly concerning given that sleep abnormalities are increasingly recognized as both a consequence of neurotoxic injury and a perpetuating factor in psychiatric morbidity among poisoning survivors. Consequently, in clinical practice, it is highly recommended to implement routine Pittsburgh Sleep Quality Index (PSQI) screenings; for patients scoring > 5, early interventions integrating cognitive-behavioral therapy and melatonin regulation should be initiated to break this vicious cycle. Concurrently, early social determinants-such as a childhood left-behind background or past traumatic exposures-serve as compounding stressors that fundamentally deplete a patient’s post-poisoning psychological resilience. Left-behind children, frequently lacking early family support, are highly susceptible to post-traumatic stress reactions following poisoning, while past major trauma acts as a continuous psychological stressor that delays the overall recovery process (39). Thus, embedding psychosocial history assessments during treatment and initiating psychiatric-assisted family system therapies are essential to rebuild their social support networks.

### Limitations

Several limitations were acknowledged, including the relatively small single-center sample size, which limited the statistical power for detecting subtle dimensional differences. The limited sample size is partly attributable to the high case fatality of diquat poisoning and the substantial stigma and recruitment barriers among survivors. Similar post-discharge follow-up studies in this population are typically small, and each enrolled participant represents a complete psychosocial assessment after a life-threatening event. While this constrains statistical power, the cohort nevertheless constitutes a rare and valuable dataset for this understudied population. Multivariable regression was not performed because the small sample size (*n* = 25) would result in an inadequate events-per-variable ratio and an unacceptable risk of overfitting; therefore, only univariate analyses with effect size estimation were conducted. Moreover, the cross-sectional nature of the follow-up precluded establishing definite causal trajectories, including the temporal sequence between sleep deprivation and HRQoL decline, and limited the tracking of dynamic symptom evolution. Future research warrants large-scale, multi-center prospective cohorts coupled with longitudinal biomonitoring, such as tracking oxidative stress indicators, to deeply explore underlying mechanisms. Additionally, interventional studies targeting psychosocial factors, including mindfulness-based therapies and structured family support programs, may offer novel directions for improving the prognosis of poisoned patients.

## Conclusion

In conclusion, this study provided a systematic overview of the long-term health and psychological outcomes in patients following diquat self-poisoning. The results indicated poor mental health with prominent anxiety and depression, highlighting an urgent need for targeted preventive measures against repeated self-harm, particularly among high-risk adolescents and young adults. These findings contributed critical evidence for designing precise clinical strategies and reducing overall medical costs.

## Data Availability

The raw data supporting the conclusions of this article will be made available by the authors, without undue reservation.
